# Cross-Comparison of Exome Analysis, Next-Generation Sequencing of Amplicons, and the iPLEX^®^ ADME PGx Panel for Pharmacogenomic Profiling

**DOI:** 10.3389/fphar.2016.00001

**Published:** 2016-01-26

**Authors:** Eng Wee Chua, Simone L. Cree, Kim N. T. Ton, Klaus Lehnert, Phillip Shepherd, Nuala Helsby, Martin A. Kennedy

**Affiliations:** ^1^Carney Centre for Pharmacogenomics, Department of Pathology, University of OtagoChristchurch, New Zealand; ^2^Faculty of Pharmacy, Universiti Kebangsaan MalaysiaKuala Lumpur, Malaysia; ^3^School of Biological Sciences, The University of AucklandAuckland, New Zealand; ^4^Auckland UniServices Sequenom Facility, Liggins Institute, The University of AucklandAuckland, New Zealand; ^5^School of Medical Sciences, The University of AucklandAuckland, New Zealand

**Keywords:** whole-exome sequencing, next-generation amplicon sequencing, multiplexed genotyping panel, variant quality score recalibration, pharmacogenomic profiling

## Abstract

Whole-exome sequencing (WES) has been widely used for analysis of human genetic diseases, but its value for the pharmacogenomic profiling of individuals is not well studied. Initially, we performed an in-depth evaluation of the accuracy of WES variant calling in the pharmacogenes *CYP2D6* and *CYP2C19* by comparison with MiSeq^®^ amplicon sequencing data (*n* = 36). This analysis revealed that the concordance rate between WES and MiSeq^®^ was high, achieving 99.60% for variants that were called without exceeding the truth-sensitivity threshold (99%), defined during variant quality score recalibration (VQSR). Beyond this threshold, the proportion of discordant calls increased markedly. Subsequently, we expanded our findings beyond *CYP2D6* and *CYP2C19* to include more genes genotyped by the iPLEX^®^ ADME PGx Panel in the subset of twelve samples. WES performed well, agreeing with the genotyping panel in approximately 99% of the selected pass-filter variant calls. Overall, our results have demonstrated WES to be a promising approach for pharmacogenomic profiling, with an estimated error rate of lower than 1%. Quality filters, particularly VQSR, are important for reducing the number of false variants. Future studies may benefit from examining the role of WES in the clinical setting for guiding drug therapy.

## Introduction

Whole-exome sequencing (WES) is an increasingly important technology in rare-disease (Maxmen, 2011) and drug-response genetics (Price et al., 2012). Its core technique comprises simultaneous capture, enrichment and sequencing of protein-coding and untranslated regions within the genome (exome). Besides being an effective tool for detecting potentially disease-causing variant(s), WES can also provide added information on variation in pharmacogenes. Though WES data has been shown to be highly accurate in previous studies, provided that appropriate quality filters are applied (Wang et al., 2013; Linderman et al., 2014; Strom et al., 2014; Yi et al., 2014), none of these studies have specifically explored the use of WES data for pharmacogenomic profiling. Notably, the accuracy of WES variant calling could be compromised by the failure of the technology to resolve highly similar genes (Drögemöller et al., 2013). In particular, sequencing of the *CYP2D6* gene is confounded by the presence of closely related pseudogenes, *CYP2D7* and *CYP2D8*, such that pre-amplification with long-range PCRs is usually applied to avoid undesired sequence contamination (Stüven et al., 1996).

The work described in this paper had two primary aims. First, we carried out an in-depth cross-validation of WES variant calls in *CYP2D6* and *CYP2C19* for 36 samples by amplicon sequencing on the MiSeq^®^ platform. Then, we expanded our findings and evaluated the more general applicability of WES to pharmacogenomic profiling by cross-comparison with the iPLEX^®^ ADME PGx Panel. The iPLEX^®^ADME PGx panel uses the MassARRAY^®^ system (Agena Bioscience, San Diego, CA, USA) to simultaneously analyze 184 single nucleotide polymorphisms (SNP), insertions and deletions (INDELs) and 16 copy number variations (CNV) across 36 genes relevant to drug absorption, distribution, metabolism, and excretion.

## Materials and Methods

### Sample Population

A total of 36 samples were included in this study. These sequenced samples comprised various research samples referred to our laboratory for pharmacogenomic investigation. This study was approved by the Southern Health and Disability Ethics Committee, New Zealand. Potential participants were contacted first by mail and were required to indicate interest to participate by filling in and returning an enclosed form. Face-to-face interviews were subsequently conducted to obtain written consent and collect relevant medical history. The study information sheet and consent form included procedures for handling of incidental findings, which would be followed up in consultation with a clinical geneticist. DNA was extracted from peripheral blood leukocytes using a KingFisher Flex Magnetic Particle Processor, as per the manufacturer’s instructions (Thermo Fisher Scientific, Waltham, MA, USA).

### High-Throughput Sequencing and Genotyping

Briefly, for all 36 samples, WES and amplicon sequencing of the *CYP2D6* and *CYP2C19* genes were performed. For WES, paired-end 100-bp sequence reads were generated on HiSeq^®^ 2000 and aligned by BWA v0.74 (Li and Durbin, 2009) to the human GRCh37.p13 reference assembly and processed with SAMtools v0.1.19 (Li et al., 2009) and Picard v1.96 (http://picard.sourceforge.net). Reads originating from PCR duplicates were removed with Picard before and after local realignment around potential indels with GATK v2.7.1 (McKenna et al., 2010). Illumina base quality scores were recalibrated with GATK in the final alignments. Per-sample identification of SNVs and indels was performed using the HaplotypeCaller algorithm in GATK (v3.3-0). Variants identified in 124 unrelated exomes were added to empower genotyping (GATK GenotypeGVCFs, v3.3-0) and variant quality score recalibration (VQSR; GATK v3.2-2; DePristo et al., 2011).

Details for processing of amplicon sequencing data are presented in Supplementary Methods. Raw sequence reads, which had a mean length of 151 bp, were first trimmed using Trimmomatic v0.30 to remove contaminating adapter-index sequences (Lohse et al., 2012). Subsequent analysis was performed using tools available on the Galaxy server (Giardine et al., 2005; Blankenberg et al., 2010; Goecks et al., 2010). Trimmed reads were aligned to a custom reference sequence using BWA-backtrack, duplicates were removed with Picard v1.56.0, then local base realignment around indels was carried out with GATK. Finally, variants were called with GATK’s Unified Genotyper v0.0.6.

A subset of twelve samples were then selected for multiplexed genotyping by the iPLEX^®^ ADME PGx Panel (Agena Bioscience, San Diego, CA, USA). DNAs from these samples were standardized to 10 ng/μL in a final volume of 200 μL. This was followed by genotyping on the MassARRAY^®^ System (Agena Bioscience) using iPLEX^®^ Gold Biochemistry and Typer v4.0 Software (Agena Bioscience).

### Validation of WES Variant Calls in *CYP2D6* and *CYP2C19* by Amplicon Sequencing

Whole-exome sequencing genotype calls having a read depth < 4 or a genotype quality score < 10 were designated “not evaluable.” GATK defines genotype quality score as “the Phred-scaled confidence that the genotype assignment is correct.” Further, to assess the effectiveness of VQSR at improving variant calling accuracy, we divided all variants into two sets, namely those that were called below the 99% truth sensitivity threshold, also designated “pass-filter,” and those that were called with surplus sensitivity (< 99.9%). These values represent varied levels of variant detection sensitivity relative to a set of known, true variants; and 99% is a commonly adopted threshold. Then, because the call-sets comprised a mix of on- and off-target variants, those sites that were distant from the target segments, defined for the TruSeq^TM^ capture kit, were apt to be poorly covered. Variant calls generated by MiSeq^®^ amplicon sequencing were required to have an approximate read depth of at least 10-fold to be considered sufficiently confident. A false-positive event was defined as the calling of the alternate allele that was determined to be absent by MiSeq^®^ amplicon or follow-up Sanger sequencing. A false-negative event was defined as the failure to detect the alternate allele(s).

### Cross-Comparison with the iPLEX^®^ ADME PGx Panel

Comparison was carried out for variant calls emitted by both WES and the iPLEX^®^ ADME PGx Panel. Again, only pass-filter WES genotype calls, i.e., having a read depth ≥ 4 and a genotype quality score ≥ 10, were considered. For the iPLEX^®^ data set, only genotype calls that had a call rate > 85%, indicating good quality, were selected for validation. The call rates were determined by the Typer 4 (Agena Bioscience) software, having assessed spectra quality related to the reported haplotype. Sanger sequencing was performed to resolve disagreement between the two platforms.

## Results

### Validation of *CYP2D6* and *CYP2C19* Variants by MiSeq^®^ Amplicon Sequencing

Whole-exome sequencing was carried out on 36 subjects referred to our laboratory for PGx analysis. We initially compared the WES data quality for two key pharmacogenes, *CYP2D6* and *CYP2C19*, with data generated by an amplicon sequencing assay we developed on the MiSeq^®^ (Illumina) platform, for the same 36 samples (Supplementary Methods; per-exon depths of coverage are presented in Supplementary Figures S1 and S2). A total of 43 variant sites, identified by WES in the *CYP2D6* and *CYP2C19* genes, were analyzed (**Table 1**); of these, 27 were called without violating the 99% truth-sensitivity threshold. WES variant calling was highly accurate for the 27 pass-filter variant sites, achieving a concordance rate of 99.60% (**Table 2**). For the 43 variant sites identified by WES a total of 943 individual WES genotype calls were generated across the 36 samples, but 202 calls were excluded for being of insufficient quality (read depth < 4 or genotype quality < 10) or because MiSeq^®^ amplicon sequencing data were not available. Of the 741 verifiable WES genotype calls, only three calls were found to be discordant with MiSeq^®^ data. None were false-positive. Sanger sequencing revealed that two of the three WES genotype calls that were discordant with MiSeq^®^ data (rs17885098 and rs3758581) were actually accurate (**Table 3**).

**Table 1:** All variants identified in *CYP2D6* and *CYP2C19* by whole-exome sequencing (WES).

Truth-sensitivity threshold defined by variant quality score recalibration		
	Below 99%	Within the tranche 99–99.9%
*CYP2C19*		-
Upstream region	**rs4986894**	
Exon 1	rs17885098	
Exon 2	rs17878459	
Intron 2	**rs12769205 *(^∗^35)***	
Exon 5	rs4244285 (*^∗^2*)	
Intron 5	**rs28399511**; **rs4417205**	
Exon 7	rs3758580; rs3758581	
Intron 7	**rs4917623**	
Intron 8	**rs12268020**	
*CYP2D6*		
Exon 1	rs72549358 *(^∗^28);* rs769258 (*^∗^35*); rs1065852 (*^∗^10*)	-
Exon 2	rs1081003	rs28371704; rs28371705
Intron 2	-	**22:42,525,227A > C**
Exon 3	rs1058164; rs78482768 (*^∗^28*); rs5030655 (*^∗^6*)	-
Intron 3	**rs3892097 *(^∗^4)***	-
Exon 4	-	rs139779104; rs150163869; rs28371713
Intron 4	**rs58440431**	**rs113889384**; **rs112568578**; **rs111564371**
Exon 5	rs5030656 *(^∗^9)*	-
Exon 6	rs16947 (*^∗^2*)	-
Intron 6	**rs28371725 *(^∗^41)***	-
Exon 7	-	rs61736517; rs1058172
Intron 7	-	**rs1985842**; **rs28578778**; **rs28371729**; **rs116917064**
Exon 8	rs28371732	-
Exon 9	rs1135840	-
Downstream region	**rs77845838**; **rs28371738**	**22:42,522,498G > A**

Off-target variant sites are **boldened**. Variants are labelled only with the star alleles that they define. Loss-of-function alleles: *CYP2C19^∗^2*, *^∗^35*, *CYP2D6^∗^4*, *^∗^6*; reduced-function alleles: *CYP2D6^∗^9*, *^∗^10*, *^∗^41*; functional allele: CYP2D6*2, *35; of unknown functional consequences: *CYP2D6^∗^28*.

**Table 2:** Overview of variant calls generated by WES for *CYP2D6* and *CYP2C19*, and validation by amplicon sequencing using the MiSeq^®^platform.

	Truth-sensitivity threshold	
	<99%	<99.9%
Variant sites	27	43
Total genotype calls	914	1476
DP < 4 or GQ < 10No-calls	149	204
No-calls	29	36
Evaluable callsMissing MiSeq^®^ data^1^	794 53	1308 59
Missing MiSeq^®^ data^1^	53	59
Total calls evaluated Harboring alternate allele(s)	741	1249
Discordant calls	201	364
False-positive(s)^2,4^	3	136
False-negative(s)^3,4^	0	118
Concordance rate	1	17
	99.60%	89.11%

*^1^Genotype calls generated by amplicon sequencing were required to have a read depth ≥ 10*.

*^2^Where alternate allele was incorrectly called*.

*^3^Where reference allele was incorrectly called*.

*^4^For discordant calls generated below the truth sensitivity threshold, the discrepancy was resolved by Sanger sequencing*.

*Abbreviations: DP, approximate read depth; GQ, genotype quality*.

**Table 3:** Further examination of three pass-filter discordant genotype calls.

Variant ID, alleles	Quality metrics (reference reads, alternate reads, genotype quality)	WES	MiSeq^®^ amplicon sequencing	Sanger sequencing
rs17885098, T/C	18, 0, 54	Homozygous reference	Heterozygous variant	Homozygous reference
rs3758581, G/A	7, 0, 18	Homozygous reference	Heterozygous variant	Homozygous reference
rs1135840, G/C	29, 19, 99	Heterozygous variant	Homozygous variant	Homozygous variant

In contrast, when the truth sensitivity threshold was relaxed to include an additional collection of 16 variant sites (< 99.9%), the rate of concordance with MiSeq^®^ data dropped considerably to 89.11% (**Table 2**). A total of 1512 calls were generated for the 43 variant positions; but quality filtering and a dearth of sufficiently confident MiSeq^®^ data (read depth < 10) for a number of sites resulted in a final comparison set of 1249 WES genotype calls. Of these, 136 mismatches were identified, representing a large increase of 133 discordant calls in relation to the more stringent call-set (truth-sensitivity threshold < 99%). The majority of these discrepant calls were false-positives.

### Cross-Comparison with the iPLEX^®^ ADME PGx Panel

We next sought to examine the concordance between WES data and a broad PGx profile of 192 nucleotide variations in 36 genes, generated on a subset of 12 subjects using the iPLEX^®^ ADME PGx Panel (Agena Bioscience). Of all 192 polymorphisms covered by the iPLEX^®^ ADME PGx panel, 184 are SNPs and INDELs. Notably, 16 variant sites were not captured by the Nextera exome capture kit; hence, these variants could not be screened by WES (**Table 4**). Only pass-filter WES genotype calls (a read depth ≥ 4 and a genotype quality score ≥ 10) were considered for comparison, which resulted in a final set of 64 variant sites that were called by both WES and the iPLEX^®^ ADME PGx panel (**Table 5**).

**Table 4:** Variants that are not captured by TruSeq^**TM**^ kit.

Chromosome position	Gene name	Variant (rsID)	Distance (base)^¥^
Chr2:234665659	*UGT1A1*	rs4124874	3110
Chr2:234676880	*UGT1A1*	rs55750087	6933
Chr2:234681059	*UGT1A1*	rs34993780	11112
Chr3:12299435	*GSTM1*	rs1065411	30851
Chr4:69418747	*UGT2B15*	rs1902023	15465
Chr4:69961912	*UGT2B7*	rs7662029	131
Chr7:99270539	*CYP3A5*	rs776746	87
Chr7:99366316	*CYP3A4*	rs35599367	41
Chr10:96521657	*CYP2C19*	rs12248560	656
Chr10:135340567	*CYP2E1*	rs2070673	150
Chr15:75038220	*CYP1A2*	rs2069514	2814
Chr15:75041917	*CYP1A2*	rs762551	4
Chr16:31105353	*VKORC1*	rs17708472	375
Chr16:31107689	*VKORC1*	rs9923231	1263
Chr22:19930121	*COMT*	rs737865	551
Chr22:42528382	*CYP2D6*	rs1080985	1349

*^¥^ Distance between the variant site and the closest TruSeq^TM^ target*.

**Table 5:** Variants identified by both WES (“pass-filter”) and the iPLEX^®^ ADME PGx Panel.

Gene name	Variant(s)
*ABCB1*	rs1045642; rs2032582; rs1128503; rs3213619
*ABCC2*	rs717620; rs2273697; rs3740066
*ABCG2*	rs2231142
*COMT*	rs165599; rs4680
*CYP1A1*	rs41279188; rs1799814
*CYP2A6*	rs1801272; rs28399433
*CYP2B6*	rs8192709; rs12721655; rs3745274
*CYP2C19*	rs4244285; rs3758581
*CYP2C8*	rs10509681; rs1058930; rs11572080
*CYP2C9*	rs1799853; rs1057910
*CYP2D6*	rs1065852; rs28371725; rs3892097; rs5030655
*DPYD*	rs1801265; rs3918290
*GSTP1*	rs1138272; rs1695
*NAT1*	rs4986782
*NAT2*	rs1208; rs1041983; rs1799929; rs1799930; rs1799931; rs1801280
*SLC15A2*	rs2293616; rs1143671; rs1143672
*SLC15A3*	rs2257212
*SLC22A1*	rs628031; rs12208357; rs2282143; rs34059508; rs34130495; rs72552763
*SLC22A2*	rs316019
*SLCO1B1*	rs2306283; rs4149056
*SLCO1B3*	rs4149117; rs7311358
*SLCO2B1*	rs2306168
*SULT1A1*	rs1801030; rs9282861
*TPMT*	rs1142345; rs1800460
*UGT2B15*	rs1902023
*UGT2B7*	rs7668258
*VKORC1*	rs7294

After eliminating all low-quality (described above) and missed calls from both panels, 719 genotype calls were included in our final analysis. Of these, eight calls at four variant sites were found to be discordant with the iPLEX^®^ ADME PGx Panel (**Table 6**), yielding a concordance rate of 98.89%. By Sanger sequencing, we confirmed that all these discordant calls were correctly genotyped based on the WES data.

**Table 6:** Variant calls found to be discordant between WES and the iPLEX^®^ ADME PGx Panel.

Variant^1,2^	Quality metrics	(reference reads, alternate reads, genotype quality)	WES	iPLEX^®^ ADME PGx Panel	Sanger sequencing
rs1902023	0, 5, 15	Heterozygous variant	Homozygous variant	Heterozygous variant
rs72552763	6, 4, 99	Heterozygous variant	Homozygous variant	Heterozygous variant
rs3740066^3^****	15, 16, 99	Heterozygous variant	Homozygous variant	Heterozygous variant
	17, 15, 99	Heterozygous variant	Homozygous variant	Heterozygous variant
rs99282861^3^****	7, 0, 18	Homozygous variant	Heterozygous variant	Homozygous variant
	8, 0, 24	Homozygous variant	Heterozygous variant	Homozygous variant
	4, 0, 12	Homozygous variant	Heterozygous variant	Homozygous variant
	13, 0, 39	Homozygous variant	Heterozygous variant	Homozygous variant

*^1^WES genotype calls were required to have a read depth ≥ 4 and a genotype quality score ≥ 10*.

*^2^Genotype calls generated by the iPLEX^®^ ADME PGx Panel were required to have a call rate > 85%*.

*^3^These discordant calls originated from different samples*.

## Discussion

Previous studies have established WES to be an effective, high-throughput variant detection tool that has been successfully used in the analysis of Mendelian disorders (Wang et al., 2013; Linderman et al., 2014; Strom et al., 2014; Yi et al., 2014). This sequencing technique constitutes a potent driving force in personalized medicine, generating genomic profiles that could also be utilized to tailor pharmacological treatment. Suppose an individual undergoes WES to aid diagnosis of an unknown condition, surely the added information on pharmacogenomic polymorphisms could be interpreted, curated and stored for guiding future drug therapy? Here we report the results of our assessment of WES as a potential tool for pharmacogenomic profiling.

We demonstrated that pass-filter WES variant calls in *CYP2D6* and *CYP2C19* were highly accurate, yielding a near-perfect degree of concordance with the MiSeq^®^ amplicon sequencing data, despite previous concerns that WES is likely to underperform in genes with closely related homologs (Drögemöller et al., 2013). The concordance rate decreased substantially to approximately 89% when the truth-sensitivity threshold was raised to 99.9%. The difference in concordance rate was rather striking, confirming previous findings which have demonstrated the effectiveness of VQSR at reducing errors in WES data (Yi et al., 2014). In the clinical setting, a highly accurate call-set is desired, albeit at the expense of variant-detection sensitivity. We recommend 99% to be the optimal cut-off that should minimize the number of erroneous genotype calls without overly compromising variant discovery.

Another interesting point is the usability of off-target variants (**Table 1** and Supplementary Figure S3). For instance, an important defective *CYP2D6* variant, rs3892097 (*^∗^4*), is located outside the capture intervals of the TruSeq^TM^ kit. Nevertheless, 100% agreement was found between WES and MiSeq^®^ amplicon sequencing data for the variant. Had strict target definitions been adopted with no interval extension or “padding,” this variant would be overlooked. Given the intrinsic mechanism of TruSeq^TM^ capture, labeling the off-target sequences as such is slightly misleading. The kit employs contiguous probes to target regions of interest; thus capture of overhanging sequences in fragmented genomic DNA is unavoidable. The current GATK practice supports a certain extent of flexibility in WES data processing to encompass variants located within the exon-intron boundaries. Off-target variants represent a valuable subset that gives added information, and this has already been reported by other investigators (Guo et al., 2012).

We then further analyzed the 20 off-target sites) with respect to their approximate read depth and distance from the nearest target segment. Coverage appeared to range from poor to good even for variants located at similar distances from TruSeq^TM^ targets, but was consistently low beyond 100 bases (<150-fold per 36 samples; Supplementary Figure S3). This suggests that a maximum distance of 100 bases may be a reasonable qualifying threshold for invoking an off-target variant site. Nonetheless, further analysis will still be required to more accurately quantify the acceptable deviation from the target regions that would maintain sufficient data quality for variant discovery; and to ascertain whether call-sets produced using different commercial capture kits, which have varied target definitions, could be combined and subjected to the same analysis pathway.

Using the truth-sensitivity threshold pre-defined above, we then assessed the broader applicability of WES to pharmacogenomic profiling by cross-comparison with the iPLEX^®^ ADME PGx Panel in a subset of 12 samples. The panel successfully detected 181 single-nucleotide variants per sample but of these, only 64 were also called by WES without violating the truth-sensitivity threshold. The majority of the WES-derived variant calls were consistent with those obtained from the iPLEX^®^ADME PGx Panel, giving a concordance rate of 98.89%. Eight discordant WES genotype calls were observed and were subsequently verified by Sanger sequencing, again confirming the accuracy of WES variants.

Overall, our results have demonstrated that as a pharmacogenomic screening tool, WES has an estimated error rate of lower than 1% for VQSR-filtered variants. This accuracy of the WES dataset reflects the increased reliability and quality of data now available using longer reads for WES analysis. The error rate would probably decrease further in the future with improved analysis software or sequencing workflows. Applying VQSR alone appears unlikely to remove all false-positive variant calls and additional filters are required. GATK Best Practice currently implements the Genotype Refinement workflow to achieve higher data quality. In this pipeline, genotypes with quality score < 20 are filtered out following VQSR. A recent study has also found that read depth ≥ 8 and genotype quality ≥ 20 are good thresholds for removing unreliable genotype calls (Carson et al., 2014). Our results suggest that less stringent cut-offs (read depth ≥ 4 and genotype quality ≥ 10) could be adopted to obtain more genotype calls, at marginal cost to the error rate.

## Limitations

Remote or non-exonic variants of functional significance are not detectable using the WES technology and this may limit its usefulness (Londin et al., 2015). For instance, -806C > T, the transcription-enhancing promoter variant of the *CYP2C19* gene, was not detected by WES. This variant is common in the Caucasian population with an allele frequency of 18% (Sim et al., 2006) and may have an important influence on clopidogrel responsiveness (Tiroch et al., 2010). Despite improved accuracy after applying the VQSR filter, 20% of the genotype calls from the WES dataset could not be analyzed due to their poor quality or low read depth limiting the use of WES as a reliable technique for clinical application to replace mutation scanning approaches. It is also worth noting that only about one-third of the 192 iPLEX^®^ variants were covered by pass-filter WES data.

This study did not examine any samples harboring *CYP2D6* hybrid alleles, which arise from large-scale *CYP2D7* conversion of the *CYP2D6* gene. Consequently, it is not clear how well BWA-backtrack and the downstream variant caller would resolve these chimeric sequences, which would contain a large number of mismatches to the *CYP2D6* reference sequence. Because BWA-backtrack is not designed to tolerate a high error rate (Li and Durbin, 2009), it is probably not able to process extensively *CYP2D7*-converted reads. The presence of hybrid alleles is therefore likely to adversely affect the quality of *CYP2D6* variant calling on WES data, and other strategies may need to be employed to circumvent this issue.

Finally, different analysis pipelines were employed to process MiSeq^®^ amplicon sequencing and WES data. This could have contributed to the observed discrepancy between the two sequencing approaches. However, we believe the effect was unlikely to have been sufficiently severe to affect the conclusions that we have drawn.

## Conclusion

We have demonstrated that WES is a promising tool in detecting pharmacogenomic variants, even for complex loci such as the *CYP2D6* gene. VQSR is an essential quality filter for the removal of likely false variant sites. Future studies should examine the adoption of WES in the clinical setting for guiding pharmacological therapy. For instance, exome analysis could be applied to subjects in whom CYP2D6 activity has been pharmacokinetically validated, to determine genotype-phenotype correlation (that could be occasionally obscured by extensive pseudogene conversion of the *CYP2D6* gene). Various practical aspects of reporting WES results should be considered, such as obtaining patient consent for storing and utilizing this piece of information, translating the WES data into an easy-to-understand format, and determining the actionability of novel reported variants.

## Author Contributions

EWC, SC, KT, and PS performed the experiments; EWC, SC, KT, and KL analyzed the sequence data; EWC and KT drafted the manuscript; SC, KL, PS, NH, and MK critically reviewed the manuscript.

## Conflict of Interest Statement

The authors declare that the research was conducted in the absence of any commercial or financial relationships that could be construed as a potential conflict of interest.
