# Supplementary material for: Cross-Comparison of Exome Analysis, Next-Generation Sequencing of Amplicons, and the iPLEX® ADME PGx Panel for Pharmacogenomic Profiling
Source: Front Pharmacol. 2016 Jan 26;7:1. doi: 10.3389/fphar.2016.00001 (PMC4726781; doi:10.3389/fphar.2016.00001)
Supplement: Supplementary file 1 [file Data_Sheet_1.DOCX]

**SUPPLEMENTARY METHODS**

**Amplifying the entire *CYP2D6* gene with long-range PCRs**

Long PCRs for the *CYP2D6* gene were performed as previously described to screen for duplication and deletion alleles (Wright *et al.* 2010). The 6.6-kb PCR products from the two duplex reactions were pooled in equal volumes for library construction.

**Amplifying small *CYP2C19* fragments with multiplex PCRs**

Because the *CYP2C19* gene has long introns it spans approximately 90 kb, so nine small amplicons covering the promoter region and all nine exons were generated with three multiplex PCRs. The compatibility between primer pairs was checked using a web-based tool, PriDimerCheck. Gibbs free energy cut-off to define a primer dimer was recommended at -7 kcal/mol, whereby a more negative value indicates a more stable dimer structure (Shen *et al.* 2010). Using this cut-off, no incompatible primer combinations were identified so primer pairs were combined based on the resultant product size and the ease of separation by agarose gel electrophoresis. Care was taken to ensure that the primers did not mask known polymorphic sites. Subsequently, the multiplex PCRs were optimised based on previously reported strategies (Henegariu *et al.* 1997). The reaction conditions for the three multiplex PCRs are shown in Supplementary Table 1. PCRs were generally carried out in 10 µL volumes containing 0.25U of TAQ-Ti Heat-Activated DNA Polymerase (Fisher Biotec, Wembley, WA, Australia), and 25-50 ng of input DNA, in the supplier’s buffer. Thermal cycling was preceded by heat-denaturation at 94°C for two minutes. As equal volumes of all PCR products were pooled prior to library preparation, care was taken to ensure that all three multiplex reactions resulted in similar overall yields.

**Preparing Nextera XT^TM^ sequencing libraries**

Sequencing libraries for 96 samples of *CYP2D6* and *CYP2C19* amplicons were constructed in a 96-well plate using the Nextera XT^TM^ Sample Preparation Kit (Illumina Inc., San Diego, CA, USA) as per the manufacturer's instructions. Briefly, this entailed “tagmentation” (simultaneous fragmentation and tagging) of amplicons, followed by PCR amplification using a combination of indexing primers unique to each sample, library normalization and pooling samples of equimolar concentrations. The resulting fragment libraries were sequenced from both ends on one lane of a MiSeq^®^ system (Illumina Inc., San Diego, CA, USA) with a read length of 2×100 nucleotides (sequencing service provider was New Zealand Genomics Ltd., Palmerston North, New Zealand). Data from a subset of 36 of these samples, for whom WES data was also generated, were used for this paper.

Supplementary Table 1. Primer combinations and reaction conditions for multiplex *CYP2C19* PCRs.

| Reaction | Primers^1^ | Sequence (5’-3’) | Fragment size (bp) | Primer concentration | Mg^2+^ concentration | Annealing temperature | Final buffer concentration |
| --- | --- | --- | --- | --- | --- | --- | --- |
| Triplex 1 | 2C19Prom3F  2C19Prom4R | AAT GAC CAG TGA AAC ATT GTG C  CAC CTT TAC CAT TTA ACC CCC | 907 | 0.2 µM | 2 mM | 62 °C | 1X |
|  | 2C19Ex23F  2C19Ex23R | AAA ATA TGA ATC TAA GTC AGG CTT AGT  GGA GAG CAG TCC AGA AAG GTC AGT GAT A | 607 | 0.3 µM |  |  |  |
|  | 2C19Ex8MF  2C19Ex8R | AGC TCA TGC CTC TTA TTA CTT CGT  GAA GGC ACA TGT AAG TTC CAA CTG A | 389 | 0.15 µM |  |  |  |
| Triplex2 | 2C19Ex6F  2C19Ex6R | AAA ACT GGC ACA AGA CAG GGA TG  AAA TTG GGA CAG ATT ACA GCT GCG | 456 | 0.4 µM | 3 mM | Touchdown: 65 °C decreased to 50 °C in 15 cycles, then continued with target temperature for another 20 cycles | 1.4X |
|  | 2C19Ex4MF  2C19Ex4R | GCT TTT AAG GGA ATT CAT AGG TAA G  AAA ATG TAC TTC AGG GCT TGG | 383 | 0.1 µM |  |  |  |
|  | 2C19Ex9F  2C19Ex9R | ATC TAC TCA TCC CTC CTA TGA TTC ACC G  ATG TGG CAC TCA ATG TAA CTA TTA TAG A | 529 | 0.35 µM |  |  |  |
| Triplex 3 | 2C19PrExF  2C19Ex1MR | TCA CGT GTT TTT TTA GGG GGT TA  TTG TAA CAT TGT ACC TCT AGG GAT AT | 905 | 0.3 µM | 3 mM | 62 °C | 1X |
|  | 2C19Ex5F  2C19Ex5R | CAA CCA GAG CTT GGC ATA TTG  TGA TGC TTA CTG GAT ATT CAT GC | 409 | 0.1 µM |  |  |  |
|  | 2C19Ex7MF  2C19Ex7R | TCT TCC TGC CTT CCT TTA TTG ATA  AGA GGG TAA GAA TCA TAC TGT GA | 521 | 0.15 µM |  |  |  |

^1^Most of the primers were previously reported (Blaisdell *et al.* 2002) except 2C19PrExF, 2C19Ex1MR, 2C19Ex4MF, 2C19Ex7MF, and 2C19Ex8MF.

**Bioinformatics analysis**

Amplicon sequencing data were processed via Galaxy (Giardine *et al.* 2005; Blankenberg *et al.* 2010; Goecks *et al.* 2010), which is a web-based collection of bioinformatics tools that provides a graphical user interface.

**Processing raw sequence reads prior to alignment**

Low-quality (average Phred-scale quality less than 15), unknown trailing (N) bases, and contaminating adapter-primer-index sequence read-throughs were trimmed from the raw FASTQ reads using Trimmomatic v0.30 (Lohse *et al.* 2012). A different sequence (to be trimmed) input FASTA file was used for each sample to account for the specific adapter-primer-index combination. Only reads that were at least 15 nucleotides long were retained. Trimmomatic v0.30 is a JAVA-based tool so sequence read trimming was done locally, whereas subsequent analysis steps were all performed on Galaxy.

**Preparing reference files for downstream analysis**

Sequences of amplified regions were extracted from GRCh37.p13 assemblies of human chromosome 22 and chromosome 10 (accession numbers NC_000010.10 for *CYP2D6*; NT_030059.13 for *CYP2C19*), and were concatenated into one contiguous reference sequence (of about 11 kb). A reference-ordered binding file in the VCF format was created to document known *CYP2D6* and *CYP2C19* variants along with their more commonly adopted names, which were based on the nomenclature system set by the Human CYP Allele Nomenclature Committee (<http://www.cypalleles.ki.se/>). This file was subsequently used in variant calling for annotation purposes. A BED file was included in all variant-calling steps to limit analysis to non-primer binding sites.

**Aligning sequence reads**

Trimmed sequence reads were aligned using the BWA-backtrack algorithm against a custom reference sequence described previously. Although it may seem counterintuitive to perform deduplication for PCR-based libraries, for which a high level of “PCR duplication” was anticipated, this was done nevertheless to minimise false-positives that may arise from optical artifacts during sequence detection. Library-level duplicates removal was done separately for each sample using Picard v1.56.0. Base realignment around indels was also performed on the sample level using the GATK (it was noted that the versions of various in-built tools available on Galaxy ranged from 0.0.1 to 0.0.6) (McKenna *et al.* 2010). Base quality score recalibration (BQSR) was omitted as this was considered neither necessary nor possible for a small dataset such as that produced from a targeted sequencing run. In particular, BQSR is expected to work better with large datasets composed of at least 100 million aligned bases, based on GATK’s recommendations.

**Variant calling and filtering**

Multi-sample variant calling was performed using GATK’s Unified Genotyper v0.0.6 and subsequently annotated with a set of recommended hard filters. The final VCF file was annotated with information from these filters as well as the rs ID and common name of a variant site (if any), permitting quick comparison with the Human CYP Allele Nomenclature Database (<http://www.cypalleles.ki.se/>). To distinguish between true and false-positive variant sites, quality by depth (QD) was used as the primary filter. Variant sites which were not compiled in the reference-ordered binding file, in other words not documented in the Human CYP Allele Nomenclature Database, and having a QD value of lower than 2.0, were removed. Sequence alignment files were visually inspected in Integrative Genomics Viewer (IGV) v2.3 for further assessment of potentially novel variants that passed the QD filter (Thorvaldsdóttir *et al.* 2013). To be judged acceptable, variant sites were required to be supported by well aligned reads with low numbers of mismatches, giving a visually clean view in IGV. Genotype calls for all variant sites were also individually evaluated based on the approximate read depth and genotype quality reported in the VCF file.

**SUPPLEMENTARY REFERENCES**

Blaisdell, J., Mohrenweiser, H., Jackson, J., Ferguson, S., Coulter, S., Chanas, B., et al. (2002). Identification and functional characterization of new potentially defective alleles of human *CYP2C19*. *Pharmacogenetics.* 12, 703-711.

Blankenberg, D., Kuster, G.V., Coraor, N., Ananda, G., Lazarus, R., Mangan, M., et al. (2010). “Galaxy: a web-based genome analysis tool for experimentalists”, in *Current Protocols in Human Genetics*, ed. J.L. Haines, Korf, B.R., Morton, C.C., Seidman, C.E., Seidman, J.G., Smith, D.R., et al. (New Jersey: John Wiley & Sons, Inc.), available from <http://onlinelibrary.wiley.com/doi/10.1002/0471142727.mb1910s89/full>.

Giardine, B., Riemer, C., Hardison, R.C., Burhans, R., Elnitski, L., Shah, P., et al. (2005). Galaxy: a platform for interactive large-scale genome analysis. *Genome Res.* 15, 1451-1455.

Goecks, J., Nekrutenko, A., Taylor, J. (2010). Galaxy: a comprehensive approach for supporting accessible, reproducible, and transparent computational research in the life sciences. *Genome Biol*. **11**:R86. doi: 10.1186/gb-2010-11-8-r86.

Henegariu, O., Heerema, N.A., Dlouhy, S.R., Vance, G.H., Vogt, P.H. (1997). Multiplex PCR: critical parameters and step-by-step protocol. *Biotechniques*. 23, 504-511.

Lohse, M., Bolger, A.M., Nagel, A., Fernie, A.R., Lunn, J.E., Stitt, M., et al. (2012). RobiNA: a user-friendly, integrated software solution for RNA-Seq-based transcriptomics. *Nucleic Acids Res.* **40**:W622-W627. doi: 10.1093/nar/gks540.

McKenna, A., Hanna, M., Banks, E., Sivachenko, A., Cibulskis, K., Kernytsky, A., et al. (2010). The Genome Analysis Toolkit: a MapReduce framework for analyzing next-generation DNA sequencing data. *Genome Res.* 20, 1297-1303.

Shen, Z., Qu, W., Wang, W., Lu, Y., Wu, Y., Li, Z., et al. (2010). MPprimer: a program for reliable multiplex PCR primer design. *BMC Bioinformatics*. **11**:143. doi: 10.1186/1471-2105-11-143.

Thorvaldsdóttir, H., Robinson, J.T., Mesirov, J.P. (2013). Integrative Genomics Viewer (IGV): high-performance genomics data visualization and exploration. *Brief. Bioinform.* 14, 178-192.

Wright, G.E.B., Niehaus, D.J.H., Drögemöller, B.I., Koen, L., Gaedigk, A., Warnich, L. (2010). Elucidation of *CYP2D6* genetic diversity in a unique African population: implications for the future application of pharmacogenetics in the Xhosa population. *Ann. Hum. Genet.* 74, 340-350.

Supplementary Figure 1. Average coverage depths for *CYP2C19* and *CYP2D6* exons across the 36 exome-sequenced samples. Interval definitions were based on those demarcated for TruSeq^TM^, in a BED file.

Supplementary Figure 2. Average coverage depths for *CYP2C19* and *CYP2D6* exons across the 36 amplicon-sequenced samples (deduplicated). Interval definitions were based on NC_000010 and NC_000022 for *CYP2C19* and *CYP2D6*, respectively.

Supplementary Figure 3. Approximate read depths for 20 off-target sites (*n* = 36) in relation to their distance from the nearest target interval defined for TruSeq^TM^; for instance, a variant site located directly adjacent to a target segment (or an exon) was deemed to have a distance of 0 base.
